# Tailoring the robust superhydrophobic silicon textures with stable photodetection properties

**DOI:** 10.1038/s41598-018-37853-4

**Published:** 2019-02-07

**Authors:** Min Hsiao, Kai-Yu Chen, Chia-Yun Chen

**Affiliations:** 1 Department of Materials Science and Engineering, National Cheng Kung University, Tainan, 70101 Taiwan; 2 Hierarchical Green-Energy Materials (Hi-GEM) Research Center, National Cheng Kung University, Tainan, 70101 Taiwan

## Abstract

Surface hydrophobicity of silicon with sound durability under mechanical abrasion is highly desirable for practical needs. However, the reported micro-pyramid/nanowires structures suffer from the saturation characteristics of contact angle at around 132 degree, which impede the promotions toward reaching the state of superhydrophobicity. The present study focuses on the realization of two-scale silicon hierarchical structures prepared with the facile, rapid and large-area capable chemical etching methods without the need of lithographic patterning. The designed structures, with the well combination of microscale inverted pyramids and nanowire arrays, dramatically lead to the increased wetting angle of 157.2 degree and contact-angle hysteresis of 9.4 degree. In addition, the robustness test reveals that these hierarchical textures possess the narrow contact-angle change of 4 degree responding to the varied pH values, and maintain a narrow deviation of 2 degree in wetting angle after experiencing the abrasion test. Moreover, the highly stable photodetection characteristics of such two-scale structures were identified, showing the reliable photocurrents with less than 3% of deviation under wide range of environmental humidity. By adopting a simple chemical treatment, the wetting control is demonstrated for reliable transition of superhydrophobicity and superhydrophilicity.

## Introduction

Superhydrophobic surfaces with extreme water-repellent properties have attracted the broad interest from both scientific and industrial communities, owing to the potential applications on water/oil separation^1^, self-cleaning^2,3^, water desalination^4^, fluid mixing^5^, and drug release^6^. Inspired by natural existing species, such as lotus leaves^7^ and cicada wings^8^, many successful examples have been realized by artificially mimicking those features for the creation of superhydrophobic surfaces. Such highly water-repellent characteristics were found to be the collective responses from combining microscale and nanoscale roughness in conjunction with low surface-energy coating or T-shaped geometries^9^. In aspect of wetting capillaries at force equilibrium, the contact angle and related sliding behaviors of nonplanar surface structures was determined by the involved Laplace pressures at composite interfaces, which is given by^10,11^, ${\rm{\Delta }}{\rm{P}}=-\,{\rm{\gamma }}\,\cos (\theta -\alpha )/R$, where γ represents the surface tension of water, θ the Young’s contact angle, α the inclination angle of structural sidewalls, R the inverse curvature of water droplet.

The equation illustrates the negative dependence of Laplace pressure for the reduced wetting of water drops with respect to the angle of sidewall inclination, where the vertical edge of taped structures turns to be prevailing for yielding superhydrophobic surfaces. To create such features, lithographic patterning is prerequisite on structure fabrication, which unfortunately introduces the manufacturing burden against their practical applications with the needs of nonplanar substrates. In addition, wetting hydrophobicity is more pronounced under the circumstances of more trapped air at composite interfaces^12^. This indicates that the promotion toward hydrophobic behaviors can be practically fulfilled by invoking two scale structures for the reduction of solid/liquid contact fraction. Therefore, in this study, we aim to investigate the shape effects of microscale taped textures in conjunction with nanowire-based features on the surface-wetting characteristics. In this regard, two types of hierarchical structures, including pyramidal and inverted-pyramidal bases in conjunction with nanowire structures are constructed with distinct wet etching techniques, respectively. Examinations of the practical aspects on their hydrophobic characteristics including testing with environmental pH values and mechanical abrasion are further performed. In addition, the stable photoelectric performances based on such combined structures under light illuminations are demonstrated.

## Material and Methods

### Fabrication of hierarchical structures

Single-crystalline (100)-oriented silicon (Si) wafers with n type were utilized as the starting materials. Prior to conducting etching process, the Si substrates were rinsed with acetone, isopropyl alcohol (IPA), and de-ionized (DI) water several times. For fabricating micro-pyramidal textures, the as-cleaned Si substrates were dipped in the mixed solutions containing 3 wt% of potassium hydroxide (KOH) and 20 vol% of IPA at 80 °C for 30 min. In addition, the inverted-pyramidal textures were prepared with a single-step copper (Cu) based metal-assisted chemical etching (MaCE) with a process modified from the literature^13,14^. In our etching process, 0.65 M of H_2_O_2_ was mixed with 0.01 M of CuSO_4_ and 1.2 M of HF under magnetic stir for 10 min and subsequently the Si substrates were immersed in the mixed solution at 40 °C for 15 min. After that, the etched samples were dipped in the concentrated HNO_3_ (65%) solution for 2 min and then rinsed with DI water in order to completely removing Cu nanoparticles from Si surfaces.

The hierarchical structures were synthesized by introducing Si nanowires directly on the two types of pyramidal-based textures. These nanowire structures were fabricated using the silver (Ag) based MaCE method^15–19^. In general, 0.02 M of AgNO_3_ and 4.8 M of HF was prepared at room temperature and then the texturized samples were dipped in the etching solutions for 1 min. Ag precipitates formed during etching reaction were completely removed by dipping the samples in the concentrated HNO_3_ for 1 min. To modulate the wetting properties of etched Si surfaces, perfluorooctane sulfonate (PFOS) functionalization was conducted by dipping samples in the 0.1% of PFOS solution for 30 min and then rinsed with DI water. These highly superhydrophobic surfaces can be transited to superhydrophilic states by dipping the samples in the diluted HF solutions (1%) for 30 s and then were immersed in the concentrated HNO_3_ for 1 min.

### Characterizations

Morphologies and chemical composition of synthesized samples were characterized with scanning electron microscope (SEM, Hitachi S-4800) and energy dispersive spectrometer (EDS, Oxford INCA), respectively. Wetting properties were measured with a Rame-Hart goniometer equipped with a charge-coupled device camera. To evaluate the stability of photodetection properties, 200-nm thick Au films were deposited using electron gun deposition processed with the defined shadow mask. The photocurrents of hierarchical-structure based sensors were measured with a typical semiconductor characterization system (Keithley 2400).

## Results and Discussion

**Figure 1:**
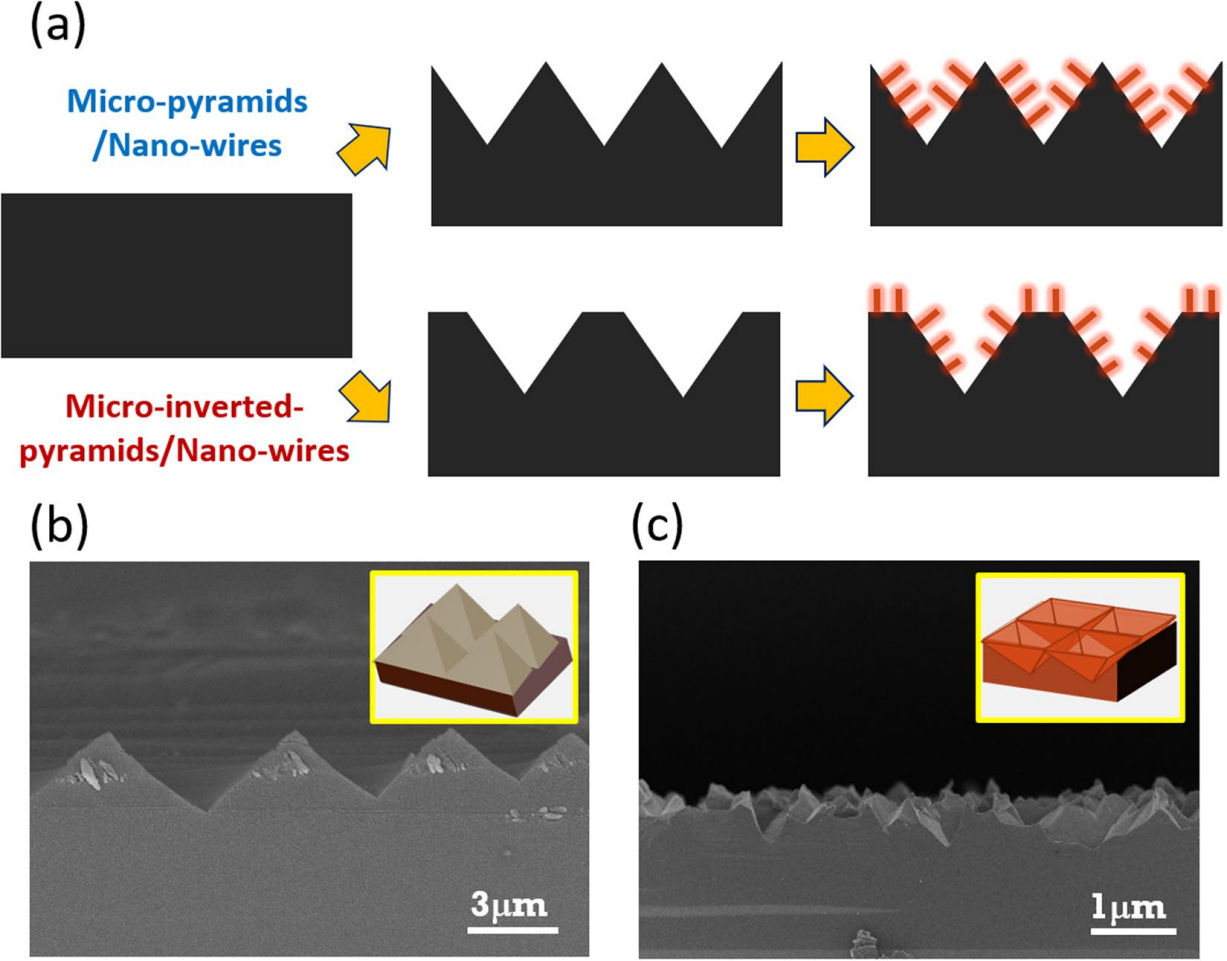
(**a**) Schematic illustrations for the formation of two distinct hierarchical structures, respectively. Cross-sectional SEM images of micro-textures: (**b**) pyramidal structures and (**c**) inverted-pyramidal structures.

Figure 1(a) presents the schematic illustrations of two distinct hierarchical Si textures employed on Si surfaces, which essentially possess the similar micro/nano combined features made by two etching steps. By applying the KOH/IPA based texturization as the first processing step, micro-pyramidal structures can be created with wide distributions of dimensions in the range of 0.6–6 μm, where the resulting textures can be found in Fig. 1(b). In the second step, dense and uniform Si nanowires are formed directly on these pre-patterned Si pyramidal structures via an Ag-based MaCE reaction. In addition, we further report an alternative micro/nano textures synthesized by two-steps chemical etching induced by two different metal nanoparticles. In the first etching step, the inverted-pyramidal structures were generated by the catalytic etching of Si assisted by Cu nanoparticles, as shown in Fig. 1(c); After carefully removing the residual Cu precipitates, Si nanowires with clear length variations along the sidewalls of inverted pyramidal structures were subsequently prepared with Ag-based MaCE process.

**Figure 2:**
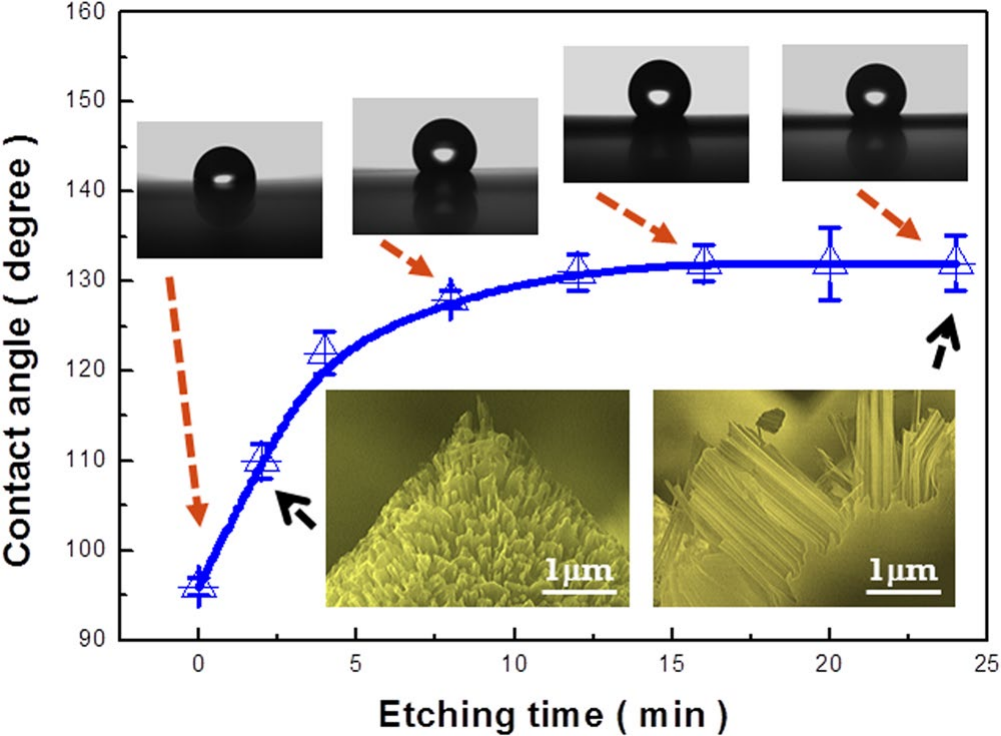
Evaluations of wetting characteristics in pyramid-based hierarchical structures. The correlated photographs of contact-angle results and cross-sectional SEM images are presented in the insert figures.

Evaluations of wetting characteristics in pyramid-based hierarchical structures are summarized in Fig. 2. From detailed investigations of contact angles in texturized samples varied by increasing the durations of Ag-based MaCE process, one can clearly observe the monolithic increase of nanowire length within the tested periods from 2 to 24 min, as demonstrated in the inserted figures of Fig. 2. In addition, the lengths of nanowires with respective to the etching time can be observed in the Supplementary Information. The introduction of nanowire textures on the pyramidal structures indeed enables to lose wetting of water droplet on surfaces, where the wetting angles of sole micro-pyramids (denoted as 0 min in Fig. 2) and the hierarchical surfaces after experiencing short-term texturiztion (2 min) are 96.3° (±1.2°) and 110.1° (±2.3°), respectively. Nevertheless, the increase of contact angle with respect to the etching durations rapidly reaches saturation correlation at 132.2° (±3.2^o^) regardless of the elongation of etching time. In addition, the contact-angle hysteresis of each case is larger than 20°. This reflects that the wetting of water droplet of such texturized surfaces without adding a low surface-energy coating cannot be completely prohibited by simply invoking long nanowires as second structure scale, which limits the practical applicability of hydrophobic wetting through these hierarchical features. It is also noted that the contact angle of such nanowire/pyramid structures can reach 156.4° (±1.5°) after introducing the PFOS coating on the surfaces.

**Figure 3:**
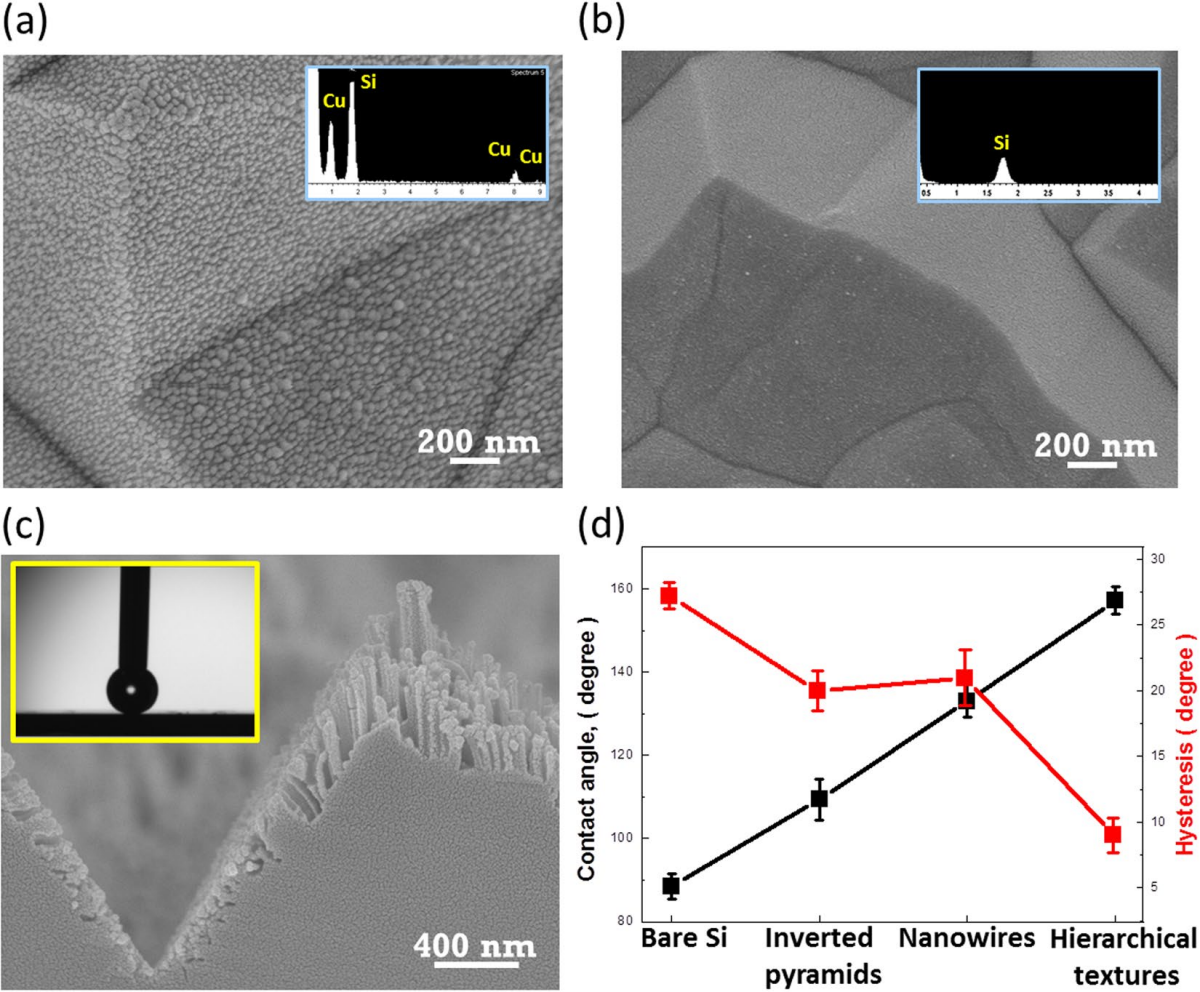
Top-view SEM images of as-prepared inverted-pyramidal structures (**a**) before and (**b**) after dissolving the Cu precipitates. The insert figures demonstrate the corresponding EDS results, respectively. (**c**) Cross-sectional SEM images of designed hierarchical structures. Contact-angle measured result is shown in the insert figure. (**d**) Plots of contact angle and related hysteresis with respect to four different textures.

To overcome the saturation characteristics that impede the promotions toward highly hydrophobic wetting, the correlated structures should be well manipulated as wetting behaviors strongly depend on the solid/liquid contact fraction introduced by the material surfaces^20^. One potential design is the employment of inverted pyramidal structures as the bases in hierarchical structures with tailored hydrophobic features. However, it has been reported that lithographic patterning on Si surfaces prior to conducing basic etching is prerequisite for the fabrication of inverted pyramidal structures^21,22^. Alternatively, here a Cu-induced MaCE method was adopted without the need of predefining substrate surfaces via a lithographic process, as detailed in the experimental section. Figure 3(a,b) compare the top-view SEM images of etched surfaces before and after removal of deposited Cu nanoparticles. These findings clearly reveal the uniform and dense deposition of Cu nanoparticles covering throughout the Si surfaces after experiencing 15-min catalytic etching (Fig. 3(a)), which identifies the fact that the anisotropic dissolution of Si is initialized by these Cu nanoparticles. The possible etching reactions can be expressed as follows,

1 $${{\rm{Cu}}}^{2+}+2{{\rm{e}}}^{-}\to {\rm{Cu}}$$

2 $${\rm{Si}}+2{{\rm{H}}}_{2}{\rm{O}}\to {{\rm{SiO}}}_{2}+4{{\rm{H}}}^{+}+4{{\rm{e}}}^{-}$$

3 $${{\rm{SiO}}}_{2}+{\rm{HF}}\to {{\rm{H}}}_{2}{{\rm{SiF}}}_{6}+{{\rm{2H}}}_{2}{\rm{O}}$$

4 $${\rm{Cu}}+{{\rm{H}}}_{2}{{\rm{O}}}_{2}+2{{\rm{H}}}^{+}\to 2{{\rm{H}}}_{2}{\rm{O}}+{{\rm{Cu}}}^{2+}$$

Etching of Si is initiated by the galvanic deposition of Cu readily on the Si surfaces. This electroless process is accompanied with the oxidation of Si underneath the as-grown Cu nanoparticles by injecting electrons to Cu^2+^ ions and followed by the removal of the oxidized Si with HF etchants. Owing to the existence of abundant H_2_O_2_ oxidant in the aqueous solutions, the deposited Cu nanoparticles suffer the rapid dissolution from H_2_O_2_ species and therefore, newly arrived Cu^2+^ ions can reach the exposed surfaces of Si that initiate another cycle of Si etching. The inverted-pyramidal like textures are gradually formed after experiencing the reaction cycles of Cu deposition and dissolution, showing the taped sidewalls along <111> orientations which can be understood by the configured backbond lattice of Si crystals. After dissolving the Cu precipitates by dipping the samples in the concentrated HNO_3_ solution for 2 min, the cleaned Si textures with complete removal of Cu nanoparticles can be found, as evidenced in the SEM image and corresponded EDS analysis (Fig. 3(b)).

The hierarchical structures with the combination of inverted pyramids and nanowires are shown in Fig. 3(c). Formation of nanowires is found to be uniformly covered on the inverted pyramidal structures, whereas the lengths of formed nanowires are unequal along the sidewalls of textures. The gradual transition of nanowires in length is clearly observed, which can be attributed to the steric hindrance of inverted-pyramid geometry that impedes the effective diffusion of AgNO_3_/HF reactive species reaching exposed Si surfaces at deep position of pyramidal sidewalls. This supports the result of etching features that the comparably long nanowires exist on the top surfaces of remained Si substrates, i.e., away from inverted-pyramid grooves, as evidenced in Fig. 3(c). Accordingly, the large contact angle of 157.2° (±3.1°) can be observed in the insert figure of Fig. 3(c), which emphasizes the structural dependence of wetting behaviors on the hierarchical structures. Moreover, comparisons of contact angle (CA) and related contact-angle hysteresis (H) are presented in Fig. 3(d). The leading hydrophobicity of tested textures is apparent to be the hierarchical structures (CA = 157.2°, H = 9.4°) involved with the incorporation of inverted pyramids at microscale and wires arrays at nanoscale. Such characteristics correlated with a large contact angle and reduced wetting hysteresis can be attributed to the two-scale structural features, where the contact lines of solid-liquid-vapor phases are segmented separately due to the well combination of inverted pyramids with nanowire arrays. Also, different from pyramidal cases, the comparably dense features of inverted pyramids acted an important rule for reducing the solid/liquid contact fraction, providing the improved dewetting phenomena. As a result, the two-scale roughened Si surfaces as well as comparably smaller features of inverted pyramids contribute to the lower solid/liquid fraction in contact with a water droplet and therefore, allowing the sound hydrophobic behaviors. This also suggests the essential role of hierarchical architectures that is responsible for controlling the hydrophobicity; whereas either the sole nanowires (CA = 136.4°, H = 21.3°) or inverted pyramids (CA = 109.4°, H = 20.0°) are incapable of reaching the superhydrophobic condition^23^. In addition, the detailed wetting results can be found in the Supplementary Information.

**Figure 4:**
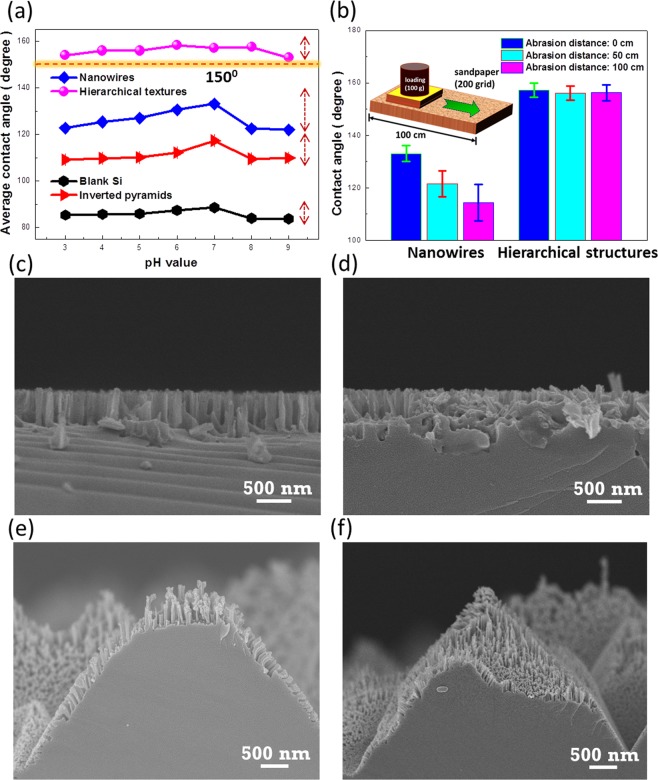
(**a**) Average contact angles of four distinct textures as a function of pH value. Contact-angle measurements were conducted after immersing the tested samples in the solutions with controlled pH values, respectively. (**b**) Abrasion test of nanowire-based and hierarchical structures. Illustration of abrasion test is schematically presented in the insert figure. Cross-sectional SEM images of nanowire textures (**c**) before and (**d**) after 100-cm abrasion test. Cross-sectional SEM images of hierarchical textures (inverted pyramids with nanowires) (**e**) before and (**f**) after 100-cm abrasion test.

Robustness of hierarchical design which consists of inverted pyramidal structures and nanowires is further examined, as demonstrated in Fig. 4(a,b). The fabricated samples were completely immersed within the aqueous solutions with various pH values ranging from 3 to 9 for 30 min, respectively, and the wetting angles were evaluated and recorded in Fig. 4(a). The consistent trends of wetting behavior with respect to the surrounding pH values can be observed from the examinations including blank Si, Si surfaces texturized with nanowires, inverted pyramids and hierarchical structures (inverted pyramids with nanowires), respectively. It has been reported the hydroxide ions may cause substitution of –H by –OH bond on Si surfaces which thereby weakens the backbond strength of Si crystal, leading them susceptible to experiencing the rupture of Si-Si backbond and the removal of surficial Si atoms^24^. This accounts for the fact that the reduction of contact angle from all of Si-related textures under basic condition (pH > 7). Nevertheless, the range of wetting-angle change (ΔCA = 4°) in hierarchical structures is explicitly narrow responding to the varied pH values, owing to their two-scale structures that both can effectively contribute to reduce the effective contact area of water droplets. In contrast, there exists a greater deviation of contact angle in nanowire (ΔCA = 9°) and micro-sized pyramids (ΔCA = 8°), respectively, which may limit their water-repellent applicability.

In addition, the mechanical abrasion of texturized samples was further tested by comparing the wetting reliability of nanowire-based and hierarchical-structure based surfaces using a linear abrasion test^25^. From the evaluations of contact angle after experiencing 0, 50 and 100-cm abrasion distance subjected to 200-grid sandpaper upon the fixed normal loading of 100 g, one can observe that the nanowire-based textures strongly lose their water-repellent capability and the remaining contact angle is reduced to 114° with substantial deviation of 18° after abrasion test for 100-cm distance upon loadings. This is because the apparent destruction of nanosized structures where the roughened topography is unable to be sustained while undergoing an abrasion contact with sandpaper, leading the water droplet to easily penetrate through and wet the surfaces. Morphologies of nanowire textures before (Fig. 4(c)) and after 100-cm abrasion (Fig. 4(d)) evidence the uneven surfaces with abundant broken nanowires appearing on the texturized surfaces, which again explains the quite large deviation of contact angle. The dramatic improvement of capability in mechanical abrasion can be found in the hierarchical structures, where the contact angle and correlated deviation maintain 155° in contact angle and 2° in contact-angle change after 100-cm abrasion. By comparing SEM observations before (Fig. 4(e)) and after abrasion (Fig. 4(f)), the two scale structures enable to prevent the complete destruction from intense abrasion forces at contact interfaces, thereby holding the surfaces in superhydrophobic state. In addition, the corresponding SEM image of sandpaper and the detailed illustrations of performing the linear abrasion test can be found in the Supplementary Information.

**Figure 5:**
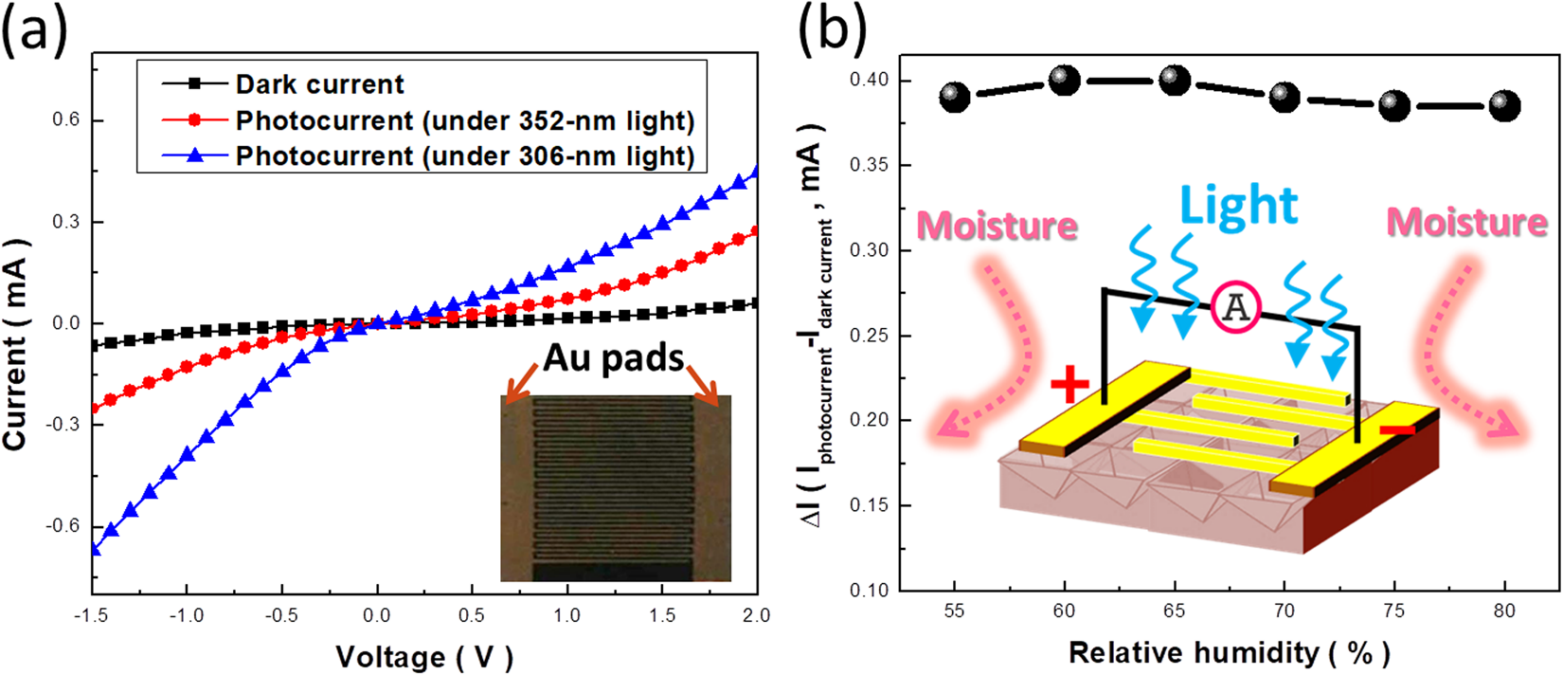
(**a**) Measured I-V characteristics of hierarchical-structure based photodetector under dark condition, illuminated with 352-nm and 306-nm lights, respectively. (**b**) Stability tests of excited photocurrents at bias voltage of 2 V under varied environmental humidity.

Based on these designed hierarchical structures with highly hydrophobic characteristics, explorations of their stable photoelectric properties are performed with the conjunction of interdigital electrodes made with 200-nm thick Au layers, as shown in the insert figure of Fig. 5(a). Accordingly, the measured current-voltage (I-V) results of the under the dark condition and light illuminations with wavelength of 352 nm and 306 nm, respectively, are presented in Fig. 5(a). Under dark condition, oxygen gases that absorb onto the surfaces of large-area n-type Si nanostructures tend to deplete the excess electrons from Si surfaces, which are therefore reduced to O_2_^−^ molecules. This accounts for the suppressed currents in the combined Si structures driven by the applied potentials because of less free negative carriers, as evidenced in Fig. 5(a). Under light illuminations with energy larger than the band gap of Si, the photogenerated electron-hole pairs are excited, and the positive holes enable to react with negatively charged O_2_^−^ captured on the Si surfaces, allowing the recovery of O_2_ molecules that becomes inactive to the carrier transport inside Si structures.

Thus, the photocurrents are dramatically increased due to the existence of photogenerated electrons with the quantity depending on the excitation energy, where the excited photocurrents at bias voltage of 2 V reach 0.46 mA under 306-nm illuminations and 0.27 mA under 352-nm illuminations, respectively, as displayed in Fig. 5(a). To examine the stability of photoelectric characteristics, the excited photocurrents at the bias voltage of 2 V are recorded in Fig. 5(b), verifying the almost unchanged photoexcited currents covering the wide range of environmental humidity from 55% to 80%. Specifically, the change of photocurrents responding to the relative humidity remains less than 3%, originating from the highly non-wetting characteristics of device surfaces that allow the minimization of undesired charge accumulation due to the chemisorption of surrounding moisture, which paves the potential way for realizing the stable and reliable photodetection properties for practical needs. By contrast, the photocurrent of the control sample with sole inverted pyramidal structures is reduced by 10% under the humidity of 70%.

**Figure 6:**
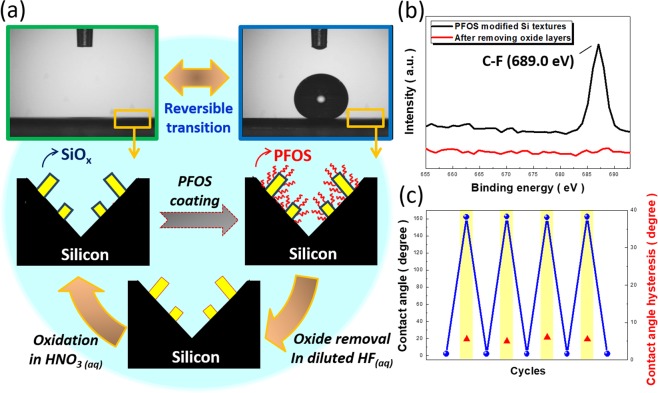
(**a**) Process flow and correlated contact-angle measured results of reversible transition between superhydrophobicity and superhydrophilicity. (**b**) XPS results of PFOS-modified hierarchical structures before and after removing oxide layers. (**c**) Cycling tests of contact angle and related hysteresis measured from hierarchical structures.

Finally, wettability change can also be employed on the designed hierarchical structures through the reversible transition between superhydrophobicity and superhydrophilicity, as demonstrated in Fig. 6(a). Surface modification of hierarchical structures with PFOS causes the remarkable superhydrophobic behaviors with contact angle of 162.1° and hysteresis of 5.0° (advancing contact angle/receding contact angle = 165.2°/160.2°) due to the fact that PFOS possesses low surface energy. According to the Cassie equation that describes the wetting characteristics of composite surface, which can be demonstrated as below^26,27^,

5 $${\cos {\rm{\theta }}}_{{\rm{c}}}={{\rm{r}}}_{{\rm{\Phi }}}{\mathrm{fcos}{\rm{\theta }}}_{{\rm{e}}}-{{\rm{r}}}_{{\rm{\Phi }}}(1-{\rm{f}})$$

where θ_e_ is the equilibrium contact angle of flat Si surfaces, θ_c_ the water droplet contact angle of rough surfaces, r_Φ_ the roughness factor and f corresponds to the area fraction of the water–air surface on Si textures. Accordingly, incorporation of low surface-energy PFOS molecules with rough hierarchical-structure bases can simultaneously enhance the surface hydrophobicity and reduce the wetting hysteresis due to the existence of trapped air between hierarchical structures and underlying water droplet. To reverse the wetting behavior of these superhydrophobic surfaces, the samples were immersed in the diluted HF (1%) for 30 s in order to dissolving the thin layer of oxide sandwiched between hierarchical bases and PFOS shells, thus completely lifting off the hydrophobic PFOS chains from the hierarchical structures. Subsequently, the surfaces of Si nanowires can be oxidized by immersing them in the HNO_3_ solution for 1 min.

Based on this simple chemical treatment, the hierarchical surfaces immediately change to superhydrophilic characteristics with extremely low contact angle (5°), as evidenced in Fig. 6(a). These findings can be further evidenced with XPS analyzed results, as shown in Fig. 6(b). In XPS spectra, the apparent C-F peak at 689.0 eV could be found on the textured surfaces while the samples were pre-treated with PFOS coating^28^. After removing a thin layer of Si oxide from the textures, the C-F peak turns to completely disappear, which identifies the removal of PFOS coating via a lift-off process and the successful modification for altering the surface wettability. To transverse back to superhydrophobicity, PFOS functionalization is employed again on the hierarchical samples, as described in the experimental details. Moreover, reversibility of transition in terms of surface wettability is tested for several cycles, where both the resulting contact angle as well as wetting hysteresis do not show any obvious changes during cycling examinations, as presented in Fig. 6(c). These results confirm the reliable transition of reversible superhydrophobicity and superhydrophilicity.

## Conclusions

In conclusion, two-scale hierarchical structures were fabricated using a facile two-steps MaCE with Cu and Ag, respectively, which enable to overcome the saturation characteristics of contact angle in conventional pyramid-based hierarchical textures. Moreover, these highly hydrophobic textures with a contact angle of 157.2° and contact-angle hysteresis of 9.4° possess both sound chemical and mechanical robust, which can be attributed to the trivial distortion after experiencing the intense abrasion forces owing to the nature of two-scale hierarchical architectures. By incorporating the designed structures with interdigital electrodes, the sound stability of photodetection properties is demonstrated, justifying the consistent excitation of photocurrents with less than 3% in variation under 55–80% of environmental humidity. In addition, the textures show the superhydrophobicity with PFOS modification, and it can be transferred to superhydrophilicity through the lifting of PFOS molecules while dissolving the underneath oxide in diluted HF solution. By conducting the cycling examinations, a sound wetting control for rapid transition of superhydrophobicity and superhydrophilicity is revealed. These results are anticipated for the potential needs of controlling wetting characteristics of Si surfaces, and can be readily applied for Si-based self-cleaning, heat-transfer treatment and functional devices.

## Supplementary information

Tailoring the robust superhydrophobic silicon textures with stable photodetection properties
