# Supplementary material for: Tailoring the robust superhydrophobic silicon textures with stable photodetection properties
Source: Sci Rep. 2019 Feb 7;9:1579. doi: 10.1038/s41598-018-37853-4 (PMC6367431; doi:10.1038/s41598-018-37853-4)
Supplement: Supplementary file 1 — Tailoring the robust superhydrophobic silicon textures with stable photodetection properties [file 41598_2018_37853_MOESM1_ESM.pdf]

Tailoring the robust superhydrophobic silicon textures with stable photodetection properties

Min Hsiao[1], Kai-Yu Chen[1], and Chia-Yun Chen[1,2*]

[1]Department of Materials Science and Engineering, National Cheng Kung University, Tainan 70101, Taiwan

[2]Hierarchical Green-Energy Materials (Hi-GEM) Research Center, National Cheng Kung University, Tainan 70101, Taiwan

S1 Relationship of nanowire length formed on the pyramids with respect to the etching time

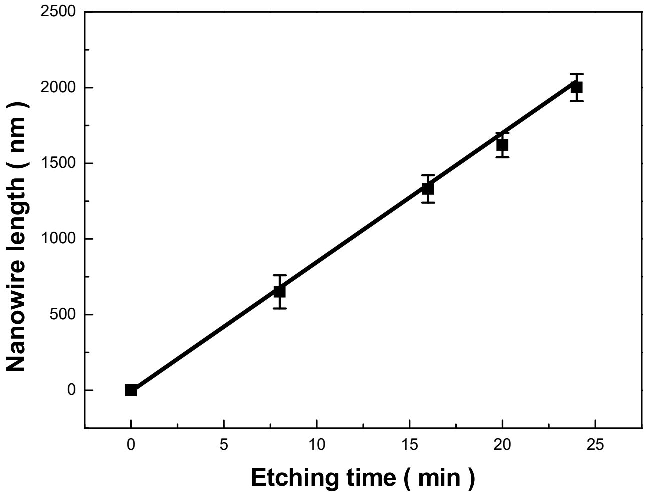

Figure S1 Plot of nanowire length versus etching time prepared with Ag-assisted chemical etching.

S2 Wetting properties of various Si-based samples

Table S1 Detailed wetting results of various samples.

	CA hysteresis	Advancing CA	Receding CA	Static CA
**Bare Si**	27.2^0	90.2^0	63.0^0	88.5^0
**Inverted pyramids**	20.0^0	114.5^0	94.5^0	109.4^0
**Nanowires**	21.3^0	136.4^0	115.1^0	133.1^0
**Hierarchical structures**	9.4^0	159.8^0	150.4^0	157.2^0

S3 Image of the sandpaper used for the linear abrasion test

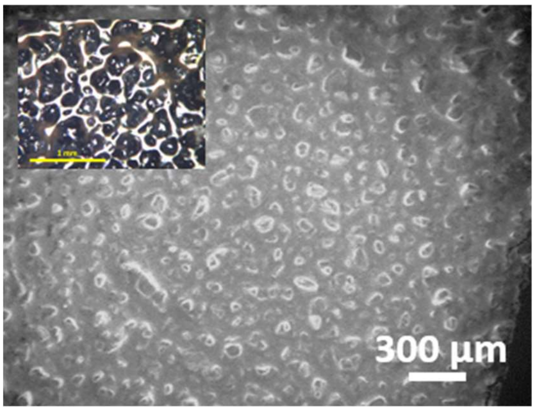

Figure S2 Top- view SEM image of sandpaper used for the linear abrasion test. The insert figure shows the corresponding optical micrograph.

S4 Illustrations of performing the linear abrasion test

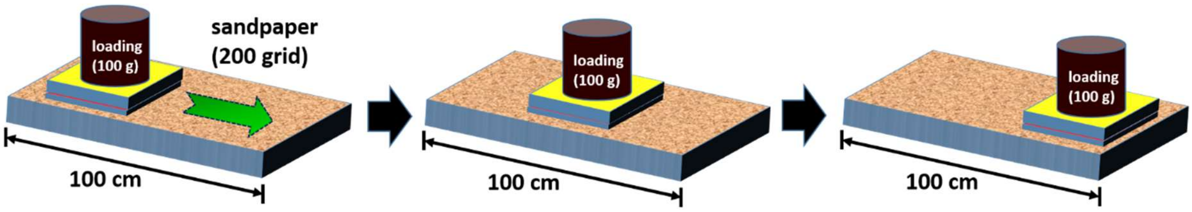

Figure S3 The linear abrasion test was performed by varying the abrasion distances under the fixed normal pressure on the sample.

S5 Wetting properties of hierarchical-structure based photodetectors.

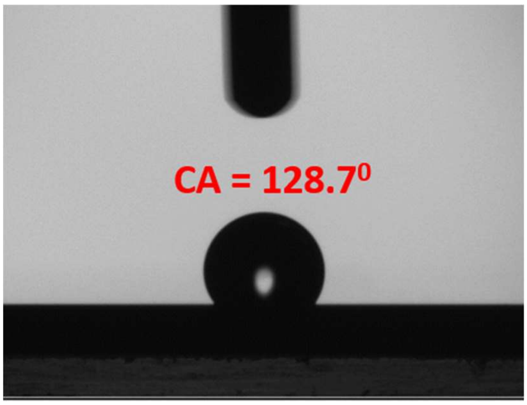

Figure S4 Contact-angle result on the surfaces of hierarchical-structure based photodetectors.
